# Unraveling the Genetic Legacy: Comparative Analysis of Yucatán Black Hairless Pig and Worldwide Indigenous Breeds

**DOI:** 10.3390/vetsci12080755

**Published:** 2025-08-13

**Authors:** Jorge Barzilai Lara-Castillo, Clemente Lemus-Flores, Job Oswaldo Bugarín-Prado, Fernando Grageola-Núñez, William Orlando Burgos-Paz

**Affiliations:** 1 Posgrado en Ciencias Biológico Agropecuarias, Universidad Autónoma de Nayarit, Tepic 63315, Mexico; 2 Unidad Académica de Medicina Veterinaria y Zootecnia, Unidad Académica de Agricultura, Universidad Autónoma de Nayarit, Tepic 63315, Mexico; clemus@uan.edu.mx (C.L.-F.); job.bugarin@uan.edu.mx (J.O.B.-P.); fgrageola@uan.edu.mx (F.G.-N.); 3 Corporación Colombiana de Investigación Agropecuaria—AGROSAVIA, Centro de Investigación Turipaná, km 13 vía Montería Cereté, Córdoba 250047, Colombia; wburgos@agrosavia.co

**Keywords:** genetic diversity, Yucatán black hairless pig, single-nucleotide polymorphism, indigenous breeds, porcine-GGP-50K, livestock

## Abstract

The Yucatán Black Hairless Pig (YBHP) is a native Mexican breed with cultural and agricultural importance in the Yucatán Peninsula. This study explored the genetic diversity and population structure of YBHP using a genome-wide single-nucleotide polymorphism (SNP) from the porcine GGP 50K chip, comparing it with native and cosmopolitan breeds worldwide. Genetic diversity analyses revealed an observed heterozygosity (HO) of 0.3602 ± 0.032 and an inbreeding coefficient (F_IS_) of 0.1517 ± 0.076, indicating moderate diversity and a relatively low level of inbreeding for an endangered native breed. ADMIXTURE analyses revealed a predominant and distinctive ancestry component exclusive to YBHP, along with shared ancestry with European breeds, particularly those of presumed Iberian origin. Principal Component Analysis (PCA) and phylogenetic network clustering supported its genomic singularity while confirming ancestral proximity to traditional European pigs. These findings highlight the dual nature of the YBHP: a genetically distinct native breed shaped by local adaptation yet retaining ancestral signals from Old World lineages. This genomic insight provides a scientific basis for conservation planning, supports the breed’s recognition as a unique genetic resource, and promotes its sustainable use in regional production systems.

## Simple Summary

The conservation of native animal breeds is essential to preserve biodiversity and ensure sustainable livestock practices. The Yucatán Black Hairless Pig (YBHP) is a unique indigenous pig breed from Mexico that is at risk of disappearing. In this study, scientists used genetic tools to evaluate how diverse and distinct this pig breed is compared to others around the world. By analyzing its DNA with a specific chip that detects genetic differences, researchers found that the YBHP has moderate genetic diversity and low levels of inbreeding. They also discovered that this breed is more closely related to European pigs than to Asian ones. These results help guide future breeding plans and support the conservation of this native pig, which plays an important role in regional culture and traditional agriculture in the Yucatán Peninsula.

## 1. Introduction

The domestication of animal species has significantly influenced human development. Pigs (Sus scrofa) were among the first species to be domesticated around 10,000 years ago in the Near East and China [1,2]. Since then, numerous breeds have emerged through both artificial selection and natural adaptation [3].

Local or adapted breeds have been defined as those “which have been in the country for a sufficient time to be genetically adapted to one or more of traditional production systems or environments in the country” [4]. Among these, local or adapted breeds are those that have evolved within traditional production systems in specific environments [4]. A subset of these, indigenous or autochthonous breeds, originates from and is uniquely adapted to particular geographic regions [5]. These native breeds often exhibit slower growth and reduced carcass yield due to fat deposition patterns and rearing conditions, distinguishing them from commercial lines [6].

The increasing dominance of high-efficiency commercial breeds has led to the genetic erosion of many local breeds, threatening their survival and reducing global genetic biodiversity [6]. Nonetheless, local pig breeds continue to serve crucial roles in rural communities—providing accessible protein sources and acting as reservoirs of adaptive traits useful for crossbreeding programs [7]. Some of these populations are considered endangered, prompting conservation initiatives [8].

The Yucatán Black Hairless Pig (YBHP) is an indigenous Mexican breed adapted to extreme tropical environments [9]. It is valued by local communities for its hairlessness—which enhances thermoregulation—disease resistance, and foraging ability [9,10]. This creole pig plays a central role in the traditional agricultural systems of the Yucatán Peninsula and has been maintained with minimal external gene flow [11].

Recent studies emphasize the urgency of preserving its genetic identity due to its potential for sustainable breeding and cultural significance [12,13]. However, little is known about its population structure or ancestral composition in a global context. Understanding these aspects is critical for guiding conservation strategies and breeding decisions.

The aim of this study was to assess the genetic diversity and structure of the YBHP using a genome-wide single-nucleotide polymorphism (SNP) and to compare it with indigenous and commercial pig breeds worldwide. Our goal was to identify the breed’s ancestral composition and unique genomic profile to support its conservation and promote its integration into structured breeding programs.

## 2. Materials and Methods

Due to the lack of a formal population census and limited pedigree records, all animals available in the region that matched the phenotypic characteristics of the Yucatán Black Hairless Pig were included. Selection criteria followed the traditional breed standards: fully black skin without white spots, absence of body hair, straight snout, and black hooves. Although producer records were limited, individuals presumed to be unrelated were prioritized to reduce sampling of close relatives. A total of 141 Yucatán Black Hairless Pigs (YBHP), comprising 26 boars and 115 sows from 49 farms located in central and eastern regions of the Yucatán Peninsula, were used. No ethical approval was required for this study, as it did not involve the collection of new biological samples. Genotypic data was obtained from the pre-existing dataset previously generated by the official breeders (Asociación Mexicana de Criadores de Cerdos de Origen Iberico de Yucatán) association [13] responsible for the management and conservation of the studied animals; these procedures adhered to ethical standards outlined in Mexican Official Regulatory Standards [14,15], ensuring the humane treatment of animals. This chip was selected based on its availability, cost-effectiveness, and validation in a broad range of pig breeds. The genotyping was performed by NEOGEN (composed of 50,967 SNPs), a well-established service provider that ensures data quality and standardized procedures, additionally facilitating consistent and reliable integration. However, it is important to note that this chip may have limitations in detecting rare or population-specific variants in underrepresented breeds, such as YBHP, due to the original design being based primarily on commercial populations.

To evaluate population structure and ancestry, publicly available SNP data from diverse local and cosmopolitan pig breeds worldwide were integrated (see Table 1) [8]. These datasets were filtered and standardized according to the methodological criteria described in their original publications. All data were merged into a unified dataset and aligned to the Sus scrofa 11.1 genome assembly. Only autosomal SNPs were retained for downstream analyses. Quality control and filtering of SNP data were performed using PLINK v1.9 [16]. SNPs with a minor allele frequency (MAF) < 0.05 (--maf 0.05), a genotyping rate < 90% (--geno 0.1), or individuals with >10% missing genotypes (--mind 0.1) were removed. Only SNPs located on autosomes (chromosomes 1–18) were retained. Genomic coordinates were updated to match the Sscrofa11.1 assembly using --update-map and --update-chr. Breeds represented by fewer than five individuals were excluded from the analysis. Genetic diversity within YBHP and other populations was assessed using the filtered SNP dataset (28,398 SNPs; 506 individuals). We calculated observed heterozygosity (HO), expected heterozygosity (HE), the inbreeding coefficient (F_IS_), and the minor allele frequency (MAF) for each population using PLINK v1.9 [16]. To explore population structure and infer historical demographic patterns (e.g., Iberian vs. commercial ancestry), a Principal Component Analysis (PCA) and ADMIXTURE analysis were conducted [17]. Linkage disequilibrium (LD) pruning was first performed with --indep-pairwise 50 5 0.2 in PLINK v1.9, retaining 9233 SNPs. PCA was conducted using PLINK v1.9 (--pca 20) and visualized in R v4.3.1 [16,18]. ADMIXTURE v1.20 [17] was used to perform unsupervised ancestry inference across values of K ranging from 1 to 30. The optimal K was selected based on the lowest 10-fold cross-validation error. Additionally, a partially supervised analysis was conducted by including reference populations with known Iberian and commercial ancestry, informed by local producer records. To visualize genetic relationships, a Neighbor-Net phylogenetic network was constructed in SplitsTree4 [19], using the Reynolds genetic distances derived from pairwise F_ST_ values. These distances were calculated in PLINK v2.0 [16] (--fst) using phenotype files to assign individuals to breed groups (--pheno). Animal sampling followed ethical guidelines established by the Secretaría de Agricultura, Ganadería, Desarrollo Rural, Pesca y Alimentación, as per NOM-062-ZOO-1999 [15]. No human subjects were involved. Generative AI was used exclusively for editing grammar and style. No AI tools were used in study design, data analysis, or interpretation.

## 3. Results

Among the populations studied, the Yucatán Black Hairless Pig (YBHP) exhibited a moderate level of genetic diversity, with an observed heterozygosity (HO) of 0.3602 ± 0.0323 and an inbreeding coefficient (F_IS_) of 0.1517 ± 0.0762, indicating a slight excess of homozygosity. Other American populations, such as OSSABAW (HO = 0.3666 ± 0.0305) and USYU (HO = 0.3745 ± 0.0387), showed similar levels of heterozygosity, with slightly lower F_IS_ values (0.1366 and 0.1181, respectively), suggesting reduced levels of inbreeding compared to YBHP. African populations, particularly KENYA, showed lower HO (0.3244 ± 0.0256) and higher F_IS_ (0.2363 ± 0.0605) than American breeds, indicating a more substantial loss of heterozygosity. Interestingly, Asian populations, such as CNLT, CNDH, and CNEH, exhibited the lowest HO values (<0.21) and moderate F_IS_ values ranging from 0.15 to 0.22, reflecting more structured or bottlenecked populations. In contrast, European breeds displayed heterogeneous diversity patterns. For instance, ITNS and ITMR showed high HO values (0.3847 and 0.3779, respectively), with relatively low F_IS_ coefficients (0.0904 and 0.1101), while breeds like HUMA and DEAS had notably high F_IS_ values (0.3099 and 0.2659) and lower HO, indicating significant inbreeding effects. The Iberian pig (ESIB), relevant to the ancestry of YBHP, exhibited a lower HO (0.1931 ± 0.0571) and moderate F_IS_ (0.077), consistent with a conserved and isolated genetic background. Among cosmopolitan commercial breeds, PIETRAIN and LW maintained moderate genetic diversity (HO = 0.3469 and 0.3383) with moderate F_IS_ values. However, HS displayed a notably high inbreeding coefficient (F_IS_ = 0.2950), suggesting intensive selective breeding (Table 2).

**Figure 1:**
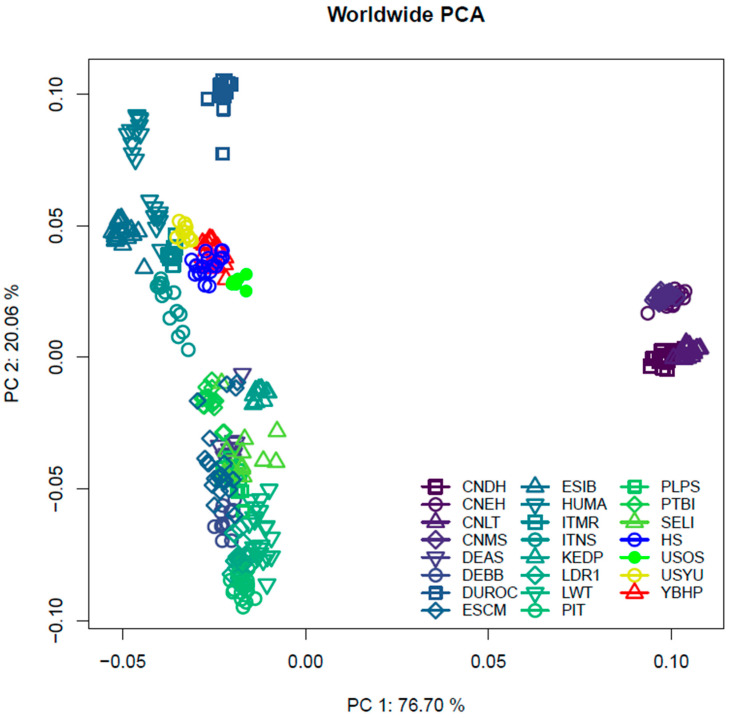
Genetic relationship based on Principal Component Analysis. For a full definition of the breeds, see Table 1.

The Principal Component Analysis (PCA) provided a spatial representation of genetic variation among populations (Figure 1). The first principal component (PC1) explained 76.70% of the total variance and separated European/American populations from Asian breeds. The second component (PC2), accounting for 20.06%, captured variation within Asian and African breeds. YBHP individuals formed a distinct yet proximal cluster near European local breeds such as ITNS, ESIB, and HUMA, while remaining separate from pure commercial breeds like LWT and PIETRAIN. This clustering pattern supports a historical genetic connection between YBHP and Iberian/European lineages, while also indicating its genomic distinctiveness. This section may be divided by subheadings. It should provide a concise and precise description of the experimental results, their interpretation, and the experimental conclusions that can be drawn.

**Figure 2:**
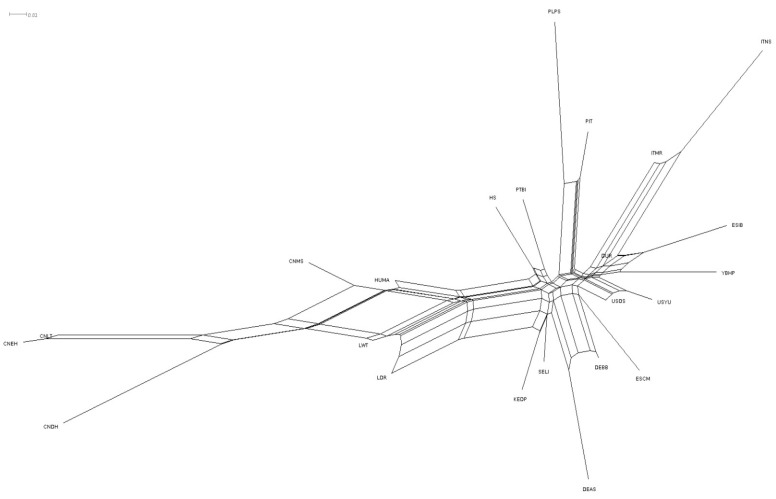
Neighbor-Net, based on Reynolds’ pairwise genetic distances among the five breed populations. For a full definition of the populations, see Table 1.

A Neighbor-Net phylogenetic network based on pairwise F_ST_ distances revealed multiple continental clusters (Figure 2). Asian breeds (CNDH, CNEH, and CNLT) were grouped tightly, reflecting close genetic similarity. European indigenous breeds (DEBB, ESIB, and DEAS) formed a second cluster, while cosmopolitan breeds (HS, LWT, and LDR) were located in a divergent group. The YBHP positioned itself intermediately between American and European populations but showed greater affinity to Iberian pigs (ESIB) than to other European or commercial groups. This placement suggests a shared ancestry, likely reflective of the historical introduction of Iberian pigs during colonial expansion.

**Figure 3:**
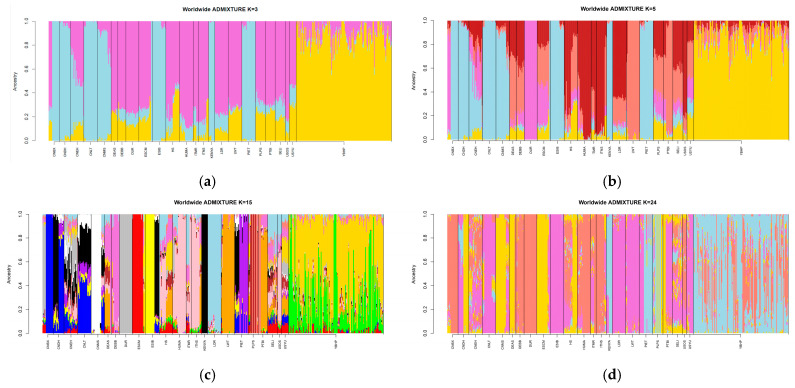
Unsupervised admixture analysis using the 9234 SNPs from our study and from the databases from [8].The breed codes are as follows: YBHP = Yucatán Black Hairless Pig; CNDH = Guangdongdahuabai; CNEH = Erhualian; CNLT = Lantang; CHMS = Meishan; DEAS = Angler Sattleschwein; DEBB = Bunte Bentheimer; ESCM = Chato Murciano; ESIB = Iberian; HUMA = Mangalica; ITMR = Mora Romagnola; ITNS = Nero Siciliano; PLPS = Pulawska; PTBI = Bisaro; SELI = Linderoth; HS = Hampshire; LDR = Landrace; LWT = LargeWhite. (**a**) Description of K = 3, (**b**) description of K = 5, (**c**) description of K = 15, and (**d**) description of K = 24.

Based on the ancestry cluster analysis, the results present the genetic admixture patterns observed across different populations for varying values of K, specifically from K = 1 to K = 25. At lower K values (K = 2 to K = 5) (Figure 3), two major ancestral components emerged: one primarily associated with Asian origins and the other with European populations, along with a third group linked to commercial breeds. For the YBHP population, the analysis at K = 5 revealed an ancestral composition consisting of 81.5% from a unique YBHP-specific component, 15.1% from a European component, 2.4% from an Asian component, and 0.8% from a commercial breed-related component.

At K = 24, which yielded the lowest cross-validation error, the YBHP retained approximately 73.51% of a unique ancestry component, indicating a strong and conserved genetic identity. Minor contributions from other populations—such as HS (1.7%), SELLI (1.6%), PTBI (0.5%), ITNS (0.2%), and ESIB (1.02%)—likely reflect historical gene flow events, including the introduction of Iberian pigs during colonization or marginal influence from commercial breeds. Despite these traces, the breed maintains its genomic distinctiveness and historical identity.

This pattern underscores the YBHP’s genetic proximity to European and Iberian breeds and supports the hypothesis of a shared historical linkage. While the YBHP shows a predominantly homogeneous structure, its genetic profile retains subtle signals of admixture with American and European populations, particularly those with Iberian origins. Regarding Asian populations, the YBHP shared only 0.033% of ancestry, and minimal affinity was observed with cosmopolitan breeds such as DUROC and LANDRACE (0.01%). These findings highlight the YBHP as a valuable genetic resource shaped by a unique combination of isolation, conservation, and historical introgression. This result aligns with the proximity observed in the PCA conducted earlier in this study, reinforcing the shared genetic background and historical connections. Overall, the DUROC, PIETRAIN, and LANDRACE breeds demonstrated the highest genomic divergence among cosmopolitan breeds, whereas the YBHP, USYU, CNEH, and ITNS populations exhibited the lowest admixture rates within indigenous breeds.

## 4. Discussion

### 4.1. Genetic Diversity Indices

Understanding genetic diversity within populations is essential for interpreting the dynamics of native and cosmopolitan breeds across the globe. In our study, the genetic diversity observed in the YBHP population closely aligns with results from previous studies on local breeds [20]. Specifically, the diversity metrics reported for YBHP are consistent with those of other populations in the Americas, corroborating findings from other authors [13,21,22,23]. In the case of YBHP, its adaptive traits such as hairlessness, disease resistance, and heat tolerance are the result of long-term selection in tropical environments. While directional selection may reduce variation at specific loci involved in these traits, the breed has maintained a moderate level of genome-wide diversity. This balance likely reflects its broad geographic range, traditional production systems, and the absence of systematic artificial selection programs, demonstrating moderate yet notable diversity despite being isolated due to the region’s rugged topography and consisting of few individuals, as it is an endangered breed [6]. Compared to the African KENYA population (HO = 0.3244; F_IS_ = 0.2363), the YBHP displays higher observed heterozygosity and lower inbreeding levels. This may reflect differences in breeding practices, where the YBHP has benefited from a broader geographic distribution and minimal artificial selection, reducing the effects of inbreeding. In contrast, the KENYA population may be subject to stronger genetic drift and founder effects, likely due to smaller and more isolated population structures. These patterns are consistent with previous reports by Babigumira and Yang [8,24], which emphasize the complex and heterogeneous genetic landscape of African livestock breeds.

The particularly low HO values observed in Asian breeds (CNDH and CNEH) may reflect the combined effects of geographic isolation and sustained artificial selection for specific traits, such as fat deposition or reproductive performance. In addition, some of these populations have undergone historical bottlenecks or have been maintained under controlled breeding programs, further reducing genetic variability. These findings align with previous reports of low heterozygosity in East Asian pigs [25,26]. The isolated nature of these Asian populations, particularly from the Chinese lineages, suggests a relatively stable gene pool with limited introgression from non-native breeds.

Similarly, the European breeds in this study presented diversity indices consistent with findings from Muñoz et al. (2019) [27], although higher than those reported by Bordonaro et al. (2023) [5], likely due to the differences in sample size and the geographic scope of the study. The cosmopolitan breeds analyzed showed genetic variability aligning with values reported by Yang [8], indicating a broad genetic base resulting from their extensive use in commercial breeding programs.

### 4.2. Population Structure

The analysis of population structure revealed distinct genetic relationships between the YBHP population and other global breeds. The YBHP breed, while geographically isolated in the Yucatán Peninsula of Mexico, exhibited a unique genetic signature that aligns with its historical development and conservation efforts. Our PCA demonstrated a genetic proximity between YBHP and European populations, specifically the ESCM and DEBB breeds, suggesting a significant European influence in its genetic makeup. This finding supports the hypothesis that European pig populations, particularly Iberian, played a pivotal role in shaping the genetic landscape of the YBHP, likely through historical trade routes during the colonization of the Americas [22,23]. The PCA results also demonstrate proximity between ESIB, ITNS, and HUMA breeds, further substantiating a European influence in its genetic composition [13]. This clustering pattern is consistent with the hypothesis of historical introgression from Iberian pigs into native populations during the colonial period [23]. The close genetic relationship between YBHP and breeds like ESIB and ITNS supports a shared ancestry originating from these introductions. In contrast, the clear separation from Asian breeds reflects the deep evolutionary divergence between the two domestication centers of pigs, Europe and Asia, which have maintained distinct genetic lineages, as described in global pig phylogeography studies [26]. This genetic affinity aligns with historical accounts suggesting that European pig breeds, particularly the Iberian lineage, played a role in shaping the genetic foundation of the YBHP [28,29].

The Asian breeds remain isolated from the rest of the populations and cluster closely among themselves in the PCA, reflecting their distinct origins. Conversely, the YBHP shows a closer genetic proximity to HS (Hampshire), a relationship that was unexpected. While this proximity does not necessarily indicate introgression, it could be explained by shared genetic traits arising from selective pressures or convergent evolutionary adaptations, such as traits favored in tropical environments [3]. Previous studies have shown that genetic proximity in PCA analyses may reflect historical parallel selection rather than actual gene flow between populations [2]. Another plausible explanation for this genetic proximity involves historical crossbreeding events between the YBHP and HS breeds, which may have occurred intentionally or inadvertently in previous years [12]. These crossings could have been aimed at improving production indices characteristic of commercial breeds, such as the number of piglets born alive. Such practices, whether deliberate or accidental, could account for the minor but detectable genetic contribution of HS to the YBHP.

Neighbor-net analysis further substantiated these findings, revealing a close genetic relationship between YBHP and the Iberian breed ESIB, with a significant genetic distance from native Asian breeds. Our analysis shows that while YBHP appears to share some genetic affinity with the Iberian population, it is clearly distinct from the Asian breeds, reinforcing the notion that there has been little genetic exchange between these two regions [8,26,30]. The genetic isolation of Asian breeds observed in this study, particularly those from China (CNEH, CNDH, and CNLT), further corroborates previous findings that reported on the preservation of distinct genetic lineages within Asian pig populations [8,31]. These studies highlighted the strong genetic identity of Chinese pig breeds, which remain largely unaltered by gene flow from European or American populations. Moreover, the positioning of YBHP as an intermediate population between the American and European clusters supports the hypothesis of shared genetic history or possible admixture between these regions [3]. This genetic affinity is supported by historical records and molecular studies indicating that Iberian pigs were introduced to the Americas during Spanish colonization, where they interbred with emerging local pig populations. This gene flow helped shape the genetic foundation of many creole breeds, including the YBHP [23,31,32].

### 4.3. Admixture Analysis

Admixture analysis for the YBHP population revealed a multi-ancestral composition with notable genetic contributions from European breeds, particularly from Iberian origins. At the optimal K-value of 25, YBHP displayed the highest ancestry proportions with Iberian breeds, such as ESIB, confirming the influence of European introgression. This ancestry pattern aligns with previous findings [13], which reported similar contributions from Iberian lineages, emphasizing the historical trade routes that facilitated genetic exchanges between these regions.

The YBHP breed also demonstrated a minor genetic affinity with cosmopolitan breeds like DUROC and PIETRAIN, with admixture levels reaching up to 0.010 for DUROC, which aligns with earlier reports of a 0.20 DUROC component in this population [12]. This suggests a degree of gene flow between local and cosmopolitan breeds, albeit minimal, indicating effective conservation practices that have limited crossbreeding in the last two decades. The genomic heterogeneity observed in YBHP supports the breed’s status as a well-preserved local breed with low inbreeding levels, reinforcing its importance in maintaining genetic diversity within Mexican pig populations [9,28].

Our study also highlighted the isolated nature of Asian breeds within the admixture framework, consistent with the findings of [26], who observed limited genetic exchanges between Asian and non-Asian pig populations. The genetic stability of these Asian breeds further underlines the geographical and cultural barriers that have historically restricted gene flow.

Previous studies suggest that pig populations in the Americas are partly of European origin [23]. Furthermore, current European pig populations are known to lack uniformity and exhibit no introgression from Asian pigs [27]. This aligns with the results of our study, where YBHP shares ancestral components with multiple European populations while remaining genetically distant from native Asian breeds.

The YBHP breed is raised in isolation on the Yucatán Peninsula, Mexico, and there is no evidence of artificial selection or crossbreeding with commercial breeds on the peninsula [22]. However, according to PCA, proximity was found with the HAMPSHIRE commercial breed and in ancestry with DUROC at a 0.010 level, acknowledging that the DUROC component was reported at 0.20 [12]; these minor genetic contributions from commercial breeds such as HS and DUROC may be attributed to unintentional or opportunistic crossbreeding practices that occurred in earlier decades, before formal conservation programs were in place. Local producers may have introduced commercial boars to improve productive traits, leading to low-level admixture. Additionally, the genetic proximity to HS may also reflect parallel selective pressures, such as those favoring adaptation to tropical environments, resulting in similar allele distributions without direct gene flow. The evidence of genomic heterogeneity and the relatively low level of inbreeding serve as proof of its effective conservation management as a local breed in the last twenty-four years.

Although the YBHP presents phenotypic traits commonly associated with adaptation to tropical conditions, such as hairlessness and environmental resilience, this study was not designed to functionally compare adaptive evolution between breeds. Therefore, we cannot determine whether the YBHP exhibits truly unique adaptive traits relative to other American native breeds or to local pig breeds in other continents. Globally, many indigenous pig populations have developed specific adaptations to their environments, often through long-term natural selection and isolation. Understanding whether the traits observed in YBHP are convergent or distinctive would require complementary approaches, such as functional genomic analyses, genome–environment association studies, or comparative transcriptomic research. These efforts would help clarify the biological mechanisms underlying adaptation and contextualize the YBHP within the broader framework of native pig breed evolution.

## 5. Conclusions

This research has provided insights into the diversity and population structure of an indigenous breed from the Yucatán Peninsula in Mexico, which is recognized as an endangered species while also serving as a significant food and economic resource for the region’s inhabitants. Genetic diversity indices demonstrated a moderate level compared to assessments of other indigenous breeds worldwide. Additionally, various statistical approaches revealed ancestral components and genetic relationships with other local breeds. Despite being an indigenous breed, it exhibited low levels of inbreeding comparable to other local and commercial breeds. This study reaffirms historical and scientific reports that previously examined the Mexican Black Hairless Pig (Pelon Mexicano) breed. The YBHP population emerges as a genetically diverse and potentially valuable resource for conservation and research purposes.

Based on the observed genetic profile, we recommend the implementation of targeted conservation strategies to prevent further genetic erosion. These include increasing the effective breeding population, avoiding consanguineous matings, and promoting community-based conservation programs. The establishment of germplasm banks and systematic monitoring of genetic diversity are also encouraged to ensure the long-term sustainability of the YBHP as a unique genetic resource.

## Figures and Tables

**Table 1:** Populations used for this study.

Breed	Country	Continent	Code	Size	Type	References
YUCATÁN BLACK HAIRLESS PIG	MEXICO	AMERICA	YBHP	20	INDIGENOUS	This Study
OSSABAW	USA	AMERICA	OSSABAW	6	INDIGENOUS	[8]
US YUCATÁN MINI PIG	USA	AMERICA	USYU	10	INDIGENOUS	[8]
KENYA LOCAL	KENYA	AFRICA	KENYA/KEDP	9	INDIGENOUS	[8]
GUANGDONGDAHUABAI	CHINA	ASIA	CNDH	20	INDIGENOUS	[8]
ERHUALIAN	CHINA	ASIA	CNEH	20	INDIGENOUS	[8]
LANTANG	CHINA	ASIA	CNLT	20	INDIGENOUS	[8]
MEIXAN	CHINA	ASIA	CHMS	20	INDIGENOUS	[8]
ANGLER SATTLESCHWEIN	GERMANY	EUROPE	DEAS	10	INDIGENOUS	[8]
BUNTE BENTHEIMER	GERMANY	EUROPE	DEBB	12	INDIGENOUS	[8]
CHATO MURCIANO	SPAIN	EUROPE	ESCM	20	INDIGENOUS	[8]
IBERIAN	SPAIN	EUROPE	IBERIAN	20	INDIGENOUS	[8]
MANGALICA	HUNGARY	EUROPE	HUMA	20	INDIGENOUS	[8]
MORA ROMAGNOLA	ITALY	EUROPE	ITMR	9	INDIGENOUS	[8]
NERO SICILIANO	ITALY	EUROPE	ITNS	15	INDIGENOUS	[8]
PULAWSKA	POLAN	EUROPE	PLPS	15	INDIGENOUS	[8]
BISARO	PORTUGAL	EUROPE	PORTUGAL	14	INDIGENOUS	[8]
LINDEROTH	SWEDEN	EUROPE	SELI	15	INDIGENOUS	[8]
DUROC	USA	COSMOPOLITAN	DUROC	20	COSMOPOLITAN	[8]
LANDRACE	DENMARK	COSMOPOLITAN	LDR/LDR1	20	COSMOPOLITAN	[8]
LARGEWHITE	DENMARK	COSMOPOLITAN	LWT	20	COSMOPOLITAN	[8]
PIETRAIN	NETHERLANDS	COSMOPOLITAN	PIT	20	COSMOPOLITAN	[8]
HAMPSHIRE	UK	COSMOPOLITAN	HS	20	COSMOPOLITAN	[8]

**Table 2:** Worldwide genetic diversity indices for the analyzed pig populations.

Population	N	MAF	HO	HE	FIS
YBHP	141	0.3325 ± 0.1997	0.3602 ± 0.0323	0.4247 ± 0001	0.1517 ± 0.0762
OSSABAW	6	0.3375 ± 0.2588	0.3666 ± 0.0305	0.4246 ± 0001	0.1366 ± 0.0718
USYU	10	0.3423 ± 0.2056	0.3745 ± 0.0387	0.4247 ± 0001	0.1181 ± 0.0913
KENYA	9	0.3398 ± 0.3882	0.3244 ± 0.0256	0.4247 ± 0.0001	0.2363 ± 0.0605
CNDH	20	0.3990 ± 0.3882	0.2035 ± 0.0118	0.4247 ± 0.0001	0.22474 ± 0.0277
CNEH	20	0.3877 ± 0.3888	0.1796 ± 0.0111	0.4247 ± 0.0001	0.15742 ± 0.0263
CNLT	20	0.4004 ± 0.3992	0.1716 ± 0.0228	0.4247 ± 0.0001	0.1595 ± 0.0537
CHMS	20	0.2468 ± 0.1476	0.3774 ± 0.0363	0.3474 ± 0.0001	-0.0862 ± 0.1043
DEAS	10	0.3277 ± 0.2628	0.3118 ± 0.0276	0.4248 ± 0.0001	0.2659 ± 0.0649
DEBB	12	0.3153 ± 0.2147	0.2775 ± 0.0449	0.4247 ± 0.0001	0.2465 ± 0.1056
ESCM	20	0.2872 ± 0.2530	0.2053 ± 0.0494	0.4247 ± 0.0001	0.1547 ± 0.016
IBERIAN	20	0.2939 ± 0.2555	0.1931 ± 0.0571	0.4247 ± 0.0001	0.077 ± 0.0135
HUMA	20	0.3098 ± 0.2141	0.2931 ± 0.0704	0.4247 ± 0.0001	0.3099 ± 0.1659
ITMR	9	0.3298 ± 0.2032	0.3779 ± 0.0102	0.4247 ± 0.0001	0.1101 ± 0.0241
ITNS	15	0.3393 ± 0.2254	0.3847 ± 0.0166	0.4247 ± 0.0001	0.0904 ± 0.0391
POLAND	15	0.3331 ± 0.2065	0.3621 ± 0.0530	0.4248 ± 0.0008	0.1476 ± 0.1249
PORTUGAL	14	0.3378 ± 0.2475	0.3374 ± 0.0481	0.4247 ± 0.0001	0.2055 ± 0.1134
SWEDEN	15	0.3111 ± 0.2444	0.2714 ± 0.0360	0.4247 ± 0.0001	0.1609 ± 0.0847
DUROC	20	0.3240 ± 0.2824	0.2854 ± 0.0208	0.4177 ± 0.0001	0.1866 ± 0.0500
LANDRACE	20	0.3308 ± 0.2472	0.3191 ± 0.0252	0.4177 ± 0.0001	0.2360 ± 0.0603
LW	20	0.3463 ± 0.2174	0.3383 ± 0.0302	0.4176 ± 0.0001	0.1900 ± 0.0723
PIETRAIN	20	0.3335 ± 0.2318	0.3469 ± 0.0165	0.4176 ± 0.0001	0.1693 ± 0.0395
HS	20	0.3078 ± 0.2363	0.2944 ± 0.0287	0.4176 ± 0.0001	0.2950 ± 0.0688

Note: number of individuals (N), average minor allele frequency (MAF), observed heterozygosity (HO), expected heterozygosity (HE), and population inbreeding coefficient (FIS).

## Data Availability

Genotyping data are owned by the Asociación Mexicana de Criadores de Cerdos de Origen Iberico de Yucatán and can be shared after signing a Material Transfer Agreement.
